# Aggregation-induced emission biomaterials for anti-pathogen medical applications: detecting, imaging and killing

**DOI:** 10.1093/rb/rbad044

**Published:** 2023-04-26

**Authors:** Zicong Zhang, Ziwei Deng, Lixun Zhu, Jialin Zeng, Xu Min Cai, Zijie Qiu, Zheng Zhao, Ben Zhong Tang

**Affiliations:** Clinical Translational Research Center of Aggregation-Induced Emission, The Second Affiliated Hospital, School of Medicine, School of Science and Engineering, Shenzhen Key Laboratory of Functional Aggregate Materials, The Chinese University of Hong Kong, Shenzhen, Guangdong 518172, China; Clinical Translational Research Center of Aggregation-Induced Emission, The Second Affiliated Hospital, School of Medicine, School of Science and Engineering, Shenzhen Key Laboratory of Functional Aggregate Materials, The Chinese University of Hong Kong, Shenzhen, Guangdong 518172, China; Clinical Translational Research Center of Aggregation-Induced Emission, The Second Affiliated Hospital, School of Medicine, School of Science and Engineering, Shenzhen Key Laboratory of Functional Aggregate Materials, The Chinese University of Hong Kong, Shenzhen, Guangdong 518172, China; Clinical Translational Research Center of Aggregation-Induced Emission, The Second Affiliated Hospital, School of Medicine, School of Science and Engineering, Shenzhen Key Laboratory of Functional Aggregate Materials, The Chinese University of Hong Kong, Shenzhen, Guangdong 518172, China; Jiangsu Co-Innovation Center of Efficient Processing and Utilization of Forest Rescources, International Innovation Center for Forest Chemicals and Materials, College of Chemical Engineering, Nanjing Forestry University, Nanjing 210037, China; Clinical Translational Research Center of Aggregation-Induced Emission, The Second Affiliated Hospital, School of Medicine, School of Science and Engineering, Shenzhen Key Laboratory of Functional Aggregate Materials, The Chinese University of Hong Kong, Shenzhen, Guangdong 518172, China; Clinical Translational Research Center of Aggregation-Induced Emission, The Second Affiliated Hospital, School of Medicine, School of Science and Engineering, Shenzhen Key Laboratory of Functional Aggregate Materials, The Chinese University of Hong Kong, Shenzhen, Guangdong 518172, China; HKUST-Shenzhen Research Institute, South Area Hi-Tech Park, Nanshan, Shenzhen, Guangdong Province 518057, China; Clinical Translational Research Center of Aggregation-Induced Emission, The Second Affiliated Hospital, School of Medicine, School of Science and Engineering, Shenzhen Key Laboratory of Functional Aggregate Materials, The Chinese University of Hong Kong, Shenzhen, Guangdong 518172, China; Department of Chemistry, Hong Kong Branch of Chinese National Engineering Research Center for Tissue Restoration and Reconstruction, The Hong Kong University of Science and Technology, Clear Water Bay, Kowloon, Hong Kong, China

**Keywords:** aggregation-induced emission, fluorescence imaging, pathogen differentiation, pathogen inactivation

## Abstract

Microbial pathogens, including bacteria, fungi and viruses, greatly threaten the global public health. For pathogen infections, early diagnosis and precise treatment are essential to cut the mortality rate. The emergence of aggregation‐induced emission (AIE) biomaterials provides an effective and promising tool for the theranostics of pathogen infections. In this review, the recent advances about AIE biomaterials for anti-pathogen theranostics are summarized. With the excellent sensitivity and photostability, AIE biomaterials have been widely applied for precise diagnosis of pathogens. Besides, different types of anti-pathogen methods based on AIE biomaterials will be presented in detail, including chemotherapy and phototherapy. Finally, the existing deficiencies and future development of AIE biomaterials for anti-pathogen applications will be discussed.

## Graphical Abstract

**Figure:**
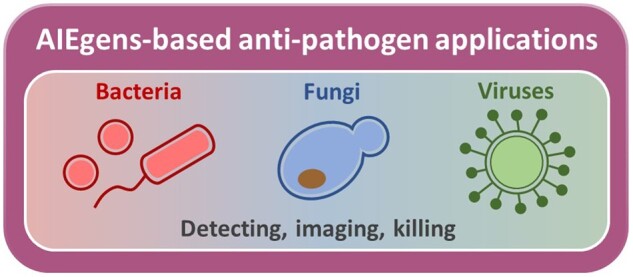

## Introduction

Human society has been plagued by infectious diseases at different stages of development [1]. It could be said that the history of human development is an epic against infectious diseases. Most infectious diseases are caused by pathogenic microbes, including bacterium, fungus and virus. In the 14th century, the Black Death caused by *Yersinia pestis*, raged for three centuries across the continent of Europe, killing more than 25 million people [2]. Since 2019, the COVID-19 pandemic caused by severe acute respiratory syndrome coronavirus 2 (SARS-CoV-2) has spread rapidly worldwide, infected nearly 700 million people and caused almost 7 million deaths [3]. Although medical level and epidemic prevention measures have been substantially improved, infectious disease is still a great threat to human health.

For pathogen infections, early diagnosis and precise treatment are essential to cut the mortality rate. At present, different technologies or methods have been developed for either fundamental research of pathogen or clinical diagnosis of pathogenic microbes, including Gram staining methods, electron microscopy (SEM, TEM, Cryo-EM) and microcolony method [4–6]. However, these techniques generally depend on expensive instruments, which also suffer the drawbacks of complicated operation, high cost, labor-intensive and time-consuming process. On the other hand, clinical treatment aims to effectively eliminate pathogens after diagnosis. Unfortunately, due to the abuse of anti-pathogen drugs (especially antibiotics), the drug-resistant pathogenic infection has become a serious global public health issue [7, 8].

With the advantages of high sensitivity and easy operation, fluorescence technology has been widely applied in biological detection and imaging [9]. However, traditional fluorescent dyes mostly suffer the influence of aggregation-caused quenching (ACQ) effect, which limited their applications and sometimes may even cause the false negative signals in detection or diagnosis [10]. In terms of these, aggregation‐induced emission (AIE) luminogens provide an excellent solution for the problems caused by ACQ materials [11]. AIE luminogens (AIEgens) indicate those luminescent materials with dim emission in solution state but enhanced emission after aggregation, the unique aggregation caused turn-on emission and enhanced photostability as well as other photoactivities (e.g. ROS: reactive oxygen species) in aggregate state make them particularly applicable for biological applications [12, 13]. After 20 years development, the application scopes of AIE materials in biological area currently have been extended to pathogens and mammalian cells related detection, tracing, therapy, theranostics etc. [14–17]. In this review, we will summarize the recent research progress of AIE biomaterials for anti-pathogen medical theranostic applications. Firstly, the working mechanism of AIEgens-based theranostics will be briefly described. Then, the applications of AIEgens for precise diagnosis of pathogens will be discussed and some representative examples will be highlighted. In addition, different types of anti-pathogen therapeutic approaches will be presented, including chemotherapy and phototherapy. At last, the existing deficiencies and future prospective of AIE biomaterials for anti-pathogen applications will be proposed.

## The overview of AIE

**Figure 1.**
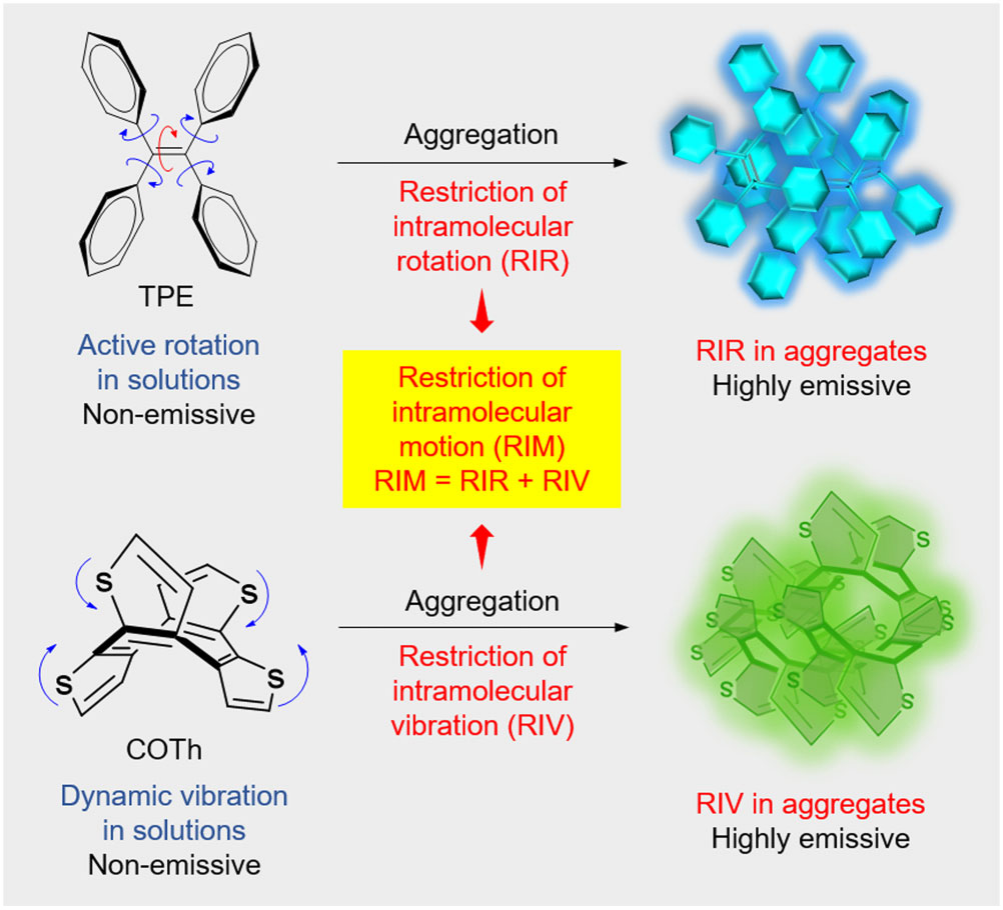
The phenomena of AIE observed in organic molecules and the mechanisms. Tetraphenylethene (TPE) is non-emissive in tetrahydrofuran (THF) solution, while it becomes highly emissive in aggregates due to the RIR. Cyclooctatetrathiophene (COTh) exhibited similar AIE property, which is caused by the restriction of RIV.

The concept of AIE was first coined by Prof. Tang in 2001, describing a photophysical phenomenon in which molecular aggregates exhibit stronger emission than the single molecules [11]. So far, the working mechanism of AIE has been well investigated, which is summarized as restriction of intramolecular motion, including restriction of intramolecular rotations (RIR) and restriction of intramolecular vibration (RIV) (Fig. 1) [13, 18]. The propeller-shaped (tetraphenylethene, TPE) or butterfly shaped (cyclooctatetrathiophene) molecules display almost no emission in dilute solutions or single molecular state, while show intensive emission in aggregate state [19, 20]. In comparison with inorganic luminescent materials and traditional ACQ luminogens, AIEgens hold many advantages, including easy-tunable chemical structure, large Stokes shift, high sensitivity in biological microenvironments, superior photostability, good biocompatibility, excellent long-term tracking capability etc. [21–24]. Thus, the fluorescent materials with AIE characteristics are excellent candidates for biological imaging and diagnostic applications [15, 25].

**Figure 2.**
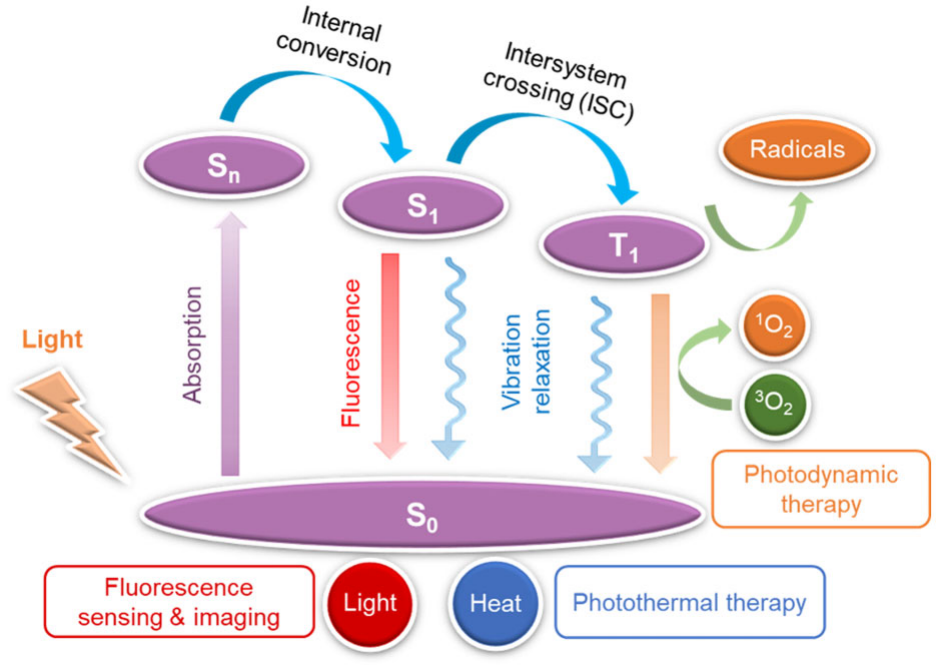
Working mechanism of PSs and photothermal agents described by the Jablonski diagram, where S and T represent the singlet and triplet states, respectively.

Apart from the superior luminescent performance in aggregate state, AIE materials also exhibit promising photodynamic and photothermal activity through regulating the intersystem crossing (ISC) efficiency and excited state molecular motion in aggregate state. The excellent photodynamic and photothermal properties of AIEgens enable their promising applications for theranostics of pathogenic infections [26–28]. The detailed working mechanism of AIE photosensitizers (PSs) and photothermal agents could be principally understood by the classical Jablonski diagram as shown in Fig. 2 [29]. After excitation from the ground state (*S*_0_) to higher excited states (*S*_n_), the exciton will decay to the lowest singlet excited state (*S*_1_) according to Kasha’s rule [30]. In general, there are three decay pathways for the excitons. For example, returning to *S*_0_ state and emitting fluorescence by a radiative pathway, which potentially could be used for fluorescence sensing and imaging [31, 32]. Additionally, the *S*_1_ can also be deactivated through non-radiative decay pathway accompanied by generating heat, which provides a pathway to develop organic photothermal agent for photothermal therapy (PTT) [33]. Besides, the excitons at *S*_1_ can also relax to the lowest triplet state (*T*_1_) through the ISC to sensitize ^3^O_2_ to generate ROS for photodynamic therapy (PDT) [34]. Since ACQ effect will quench the singlet excited state, the ROS efficiency of ACQ PSs thus are strongly suppressed in the aggregate state [35]. As a contrast, the ROS efficiency of AIE PSs do not suffer the ACQ effect but exhibit an enhancement upon aggregation due to the suppression of non-radiative decay [15, 36].

## AIE biomaterials for pathogens diagnosis

### AIE biomaterials for bacteria differentiation

**Figure 3.**
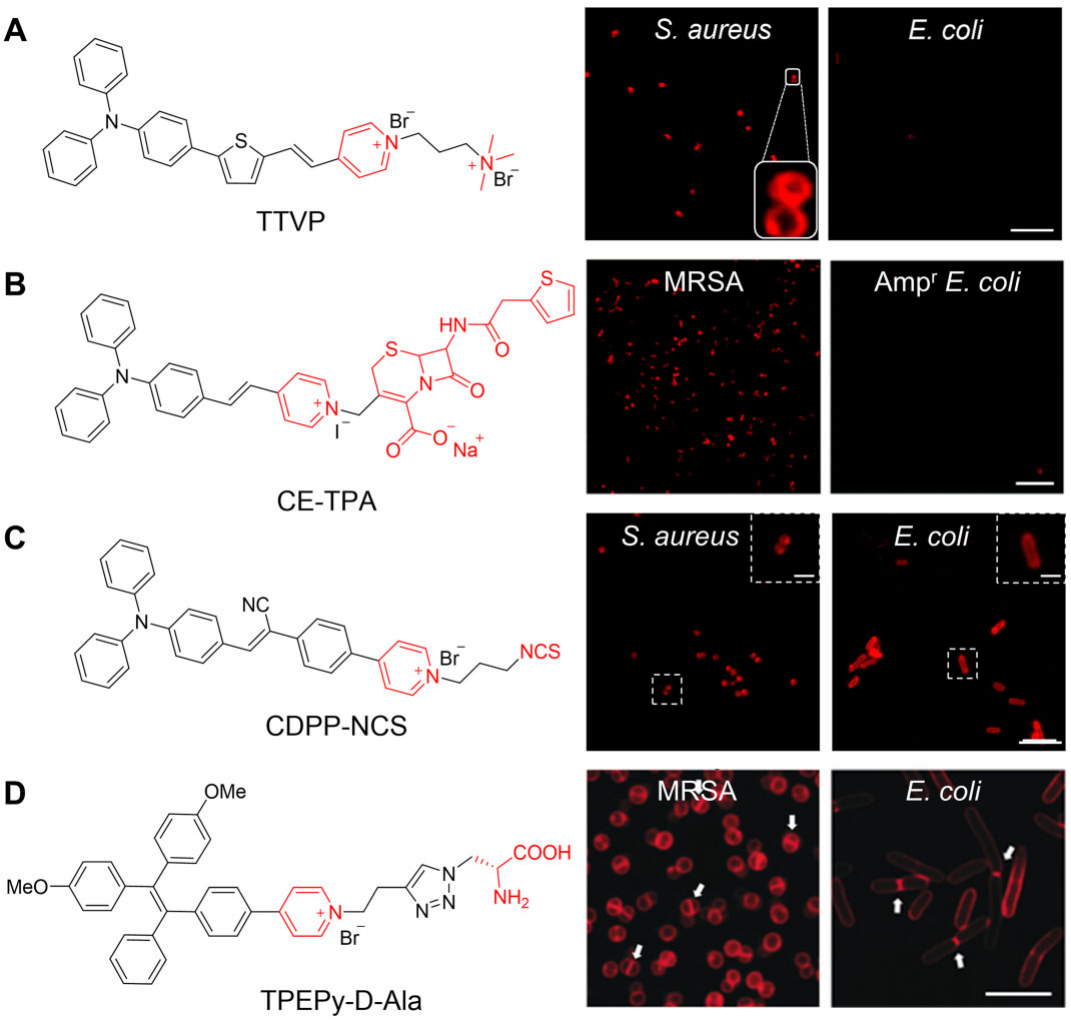
Different bacteria labeling strategies. (**A**) Charge-based labeling strategy. Scale bar: 10 μm. Reproduced from Ref. [41] with permission of Elsevier, © 2019. (**B**) Receptor-targeting labeling strategy. Scale bar: 10 μm. Reproduced from Ref. [34] with permission of the Royal Society of Chemistry, © 2022. (**C**) Isothiocyanate-based clickable covalent labeling strategy. Scale bar: 10 μm. Inset: scale bar: 2 μm. Reproduced from Ref. [47] with permission of Elsevier, © 2022. (**D**) Metabolic labeling strategy. Scale bar: 5 μm. Reproduced from Ref. [48] with permission of Wiley-VCH, © 2019.

Various bacteria exist in nature [37]. In general, bacteria can be divided into two groups by Gram staining, which is a common technique used to differentiate from their cell wall constituents [4]. Compared with Gram staining, fluorescence imaging is considered to be more sensitive and convenient for detection and differentiation . To date, AIE biomaterials have been widely utilized for bacterial imaging based on strategies summarized as follows. The most universal strategy is to introduce positive charges into AIE biomaterials to bind the bacterial envelope through electrostatic interaction [38], since the lipopolysaccharides, teichoic acid and peptidoglycan in the bacterial cell wall were all reported to be negatively charged [39, 40]. Michelle *et al*. [41] designed an AIE probe of TTVP with two positive charges (Fig. 3A). TTVP could selectively target Gram-positive bacteria *S. aureus* through a washing-free procedure with only 3 s of incubation period, implying its ultrafast bacterial discrimination feature. Furthermore, a receptor-targeting labeling strategy is introduced since various receptors exist on the bacterial envelope [42, 43]. Wang *et al*. [44] developed a receptor-targeting AIE PS (CE-TPA) by conjugating cephalothin (a β-lactam antibiotic) with a cationic AIE probe and the probe could target penicillin-binding proteins, which is the main target of β-lactam antibiotics (Fig. 3B). As a result, CE-TPA could target Gram-positive methicillin-resistant *Staphylococcus aureus* (MRSA). However, the probe failed to stain ampicillin-resistant *Escherichia coli* (Amp^r^*E. coli*) due to the denser cell wall of Gram-negative bacteria. To achieve more stable and durable labeling, covalent labeling strategies like clickable and metabolic strategies have been developed in recent years [45, 46]. An AIE probe modified with isothiocyanate (NCS), CDPP-NCS, was utilized for covalent labeling through click reaction between NCS groups and amino groups (Fig. 3C) [47]. It is notable that the probe could stably retain on the envelope for over 48 h without leakage, enabling the monitoring of interaction between bacterium and macrophage, which benefits the study of the infection process. In addition, metabolic strategies can be applied as well [48]. d-Alanine (d-Ala) could be selectively utilized by bacteria for its significant role in the synthesis of peptidoglycan. A bacteria-metabolizable AIE probe TPEPy-d-Ala that consists of d-alanine and AIE fluorophore was thus developed for fluorescence turn-on imaging of intracellular bacteria in living host cells (Fig. 3D). Notably, the septal planes of bacteria showed stronger signals owing to the ongoing peptidoglycan synthesis (the white arrows in Fig. 3D). Established on the above strategies, AIE probes have been widely applied for bacterial imaging.

**Figure 4.**
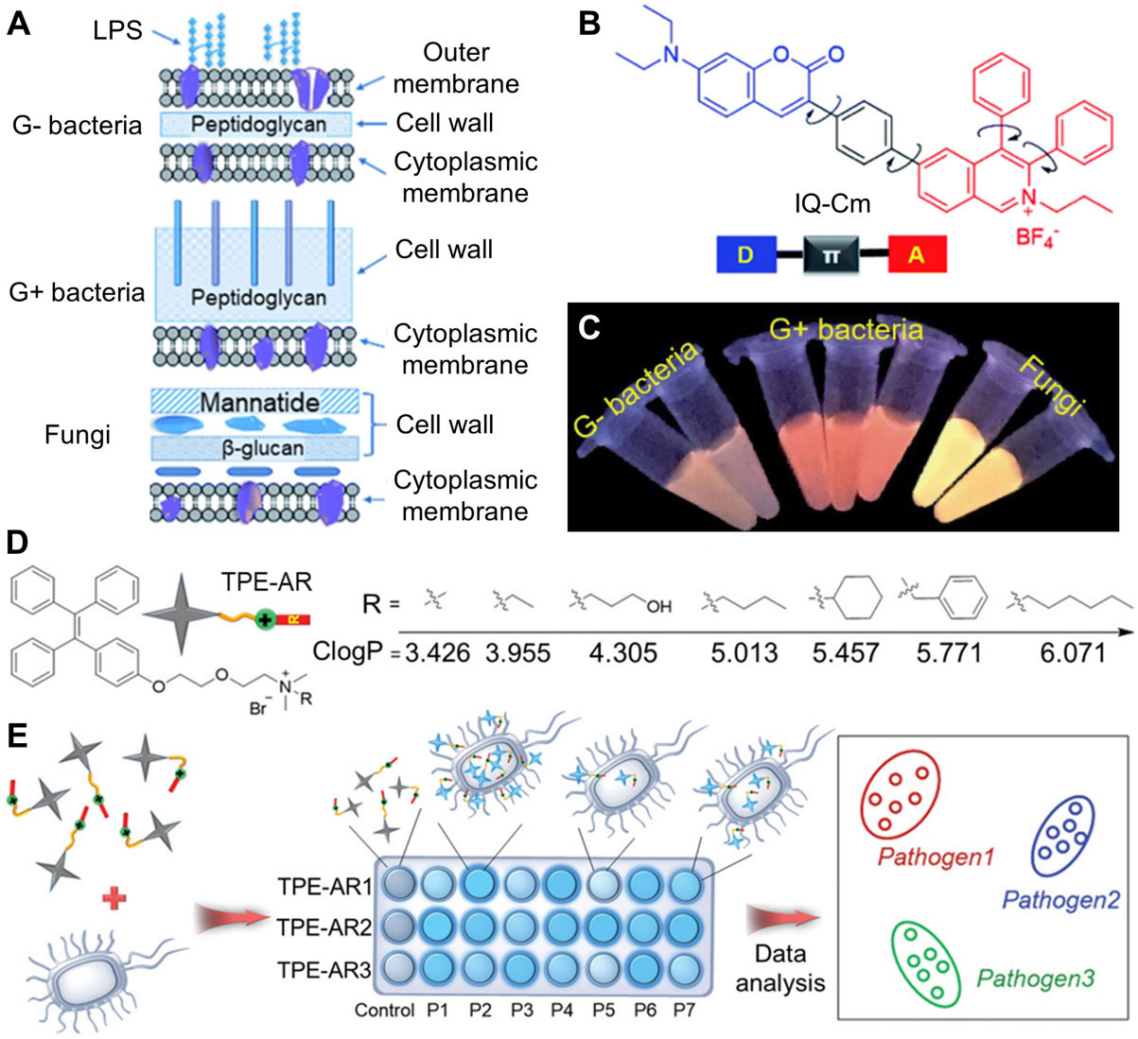
Differentiation of pathogens. (**A**) Schematic of cell envelope structures of gram-negative (G−) bacteria, gram-positive (G+) bacteria and fungi. (**B**) Chemical structure of IQ–Cm, an AIEgen with TICT characteristics. (**C**) Photographs of IQ–Cm with different pathogens in PBS solutions obtained under 365 nm irradiation. Reproduced from Ref. [55] with permission of Royal Society of Chemistry, © 2020. (**D**) Structure of TPE-ARs with various Clog*P* values. (**E**) Schematic illustration of a sensor array composed of three TPE-ARs to achieve pathogen identification. P1–P7 represent seven kinds of pathogens. Reproduced from Ref. [56] with permission of Wiley-VCH, © 2018.

The selective imaging capability of AIEgens endows them more interesting applications. Zhao *et al*. [49] designed a TPE derivative modified with boric acid (TPE-2BA) for monitoring bacterial viability, which potentially could be used for the evaluation of bacterial drug sensitivity. Interestingly, TPE-2BA can show strong fluorescence via passing through the compromised membrane of dead bacteria and binding with DNA inside. Differentiation of bacteria is one of the most important issues for rapid diagnosis and precise treatment in clinic [50–52]. It is reported that the cell envelope structures of Gram-negative (G−) bacteria, Gram-positive (G+) bacteria and fungi are different (Fig. 4A) [53, 54]. Zhou *et al*. [55] successfully developed a microenvironment-sensitive AIE probe (IQ–Cm) for fast discrimination of G− bacteria, G+ bacteria and fungi by naked eyes (Fig. 4B and C). IQ–Cm has a twisted donor–acceptor structure and shows twisted intramolecular charge transfer (TICT) characteristics with a sensitive fluorescence color response to the microenvironment of pathogens. Driven by the intrinsic structural distinctions of the above-mentioned pathogens, the positive-charged IQ–Cm can stain the pathogens and selectively locate in different sites, leading to the naked-eye discernible emission colors. Therefore, IQ–Cm can directly discriminate the three pathogens via fluorescence, providing a promising platform for rapid pathogen detection and point-of-care diagnosis.

To achieve more rapid and reliable differentiation of pathogens, a series of simple and reliable sensor arrays based on TPE derivatives (TPE-ARs) are successfully developed by Zhou *et al*. [56] for high-throughput pathogens screening. As shown in Fig. 4D, all TPE-ARs had a cationic ammonium unit but different calculated oil–water separation coefficient (Clog*P*, *n*-octanol/water partition coefficient) (3–7), which correlated with their hydrophobic side chains. The Clog*P* value could be used to evaluate the electrostatic and hydrophobic interaction between AIE probes and pathogens. Each TPE-AR shows fluorescence response after incubation with different pathogens due to the specific interaction and vice versa. Then, the fluorescence pattern was analyzed by linear discriminant analysis, followed by identification results (Fig. 4E). These sensor arrays can identify diverse pathogens, even normal and drug-resistant bacteria or blends of pathogens, with nearly 100% accuracy. This system verifies that the AIE probes can perfectly meet the actual demand of the high throughput clinical screening and medical observation. Additionally, a more sensitive high-throughput sensor array composed of TPE derivatives with varied numbers of cationic side chains was constructed recently, which could detect and discriminate various bacteria at low concentrations down to 1 × 10^3^ colony-forming unites (CFU)/ml.

### AIE biomaterials for fungi imaging

**Figure 5.**
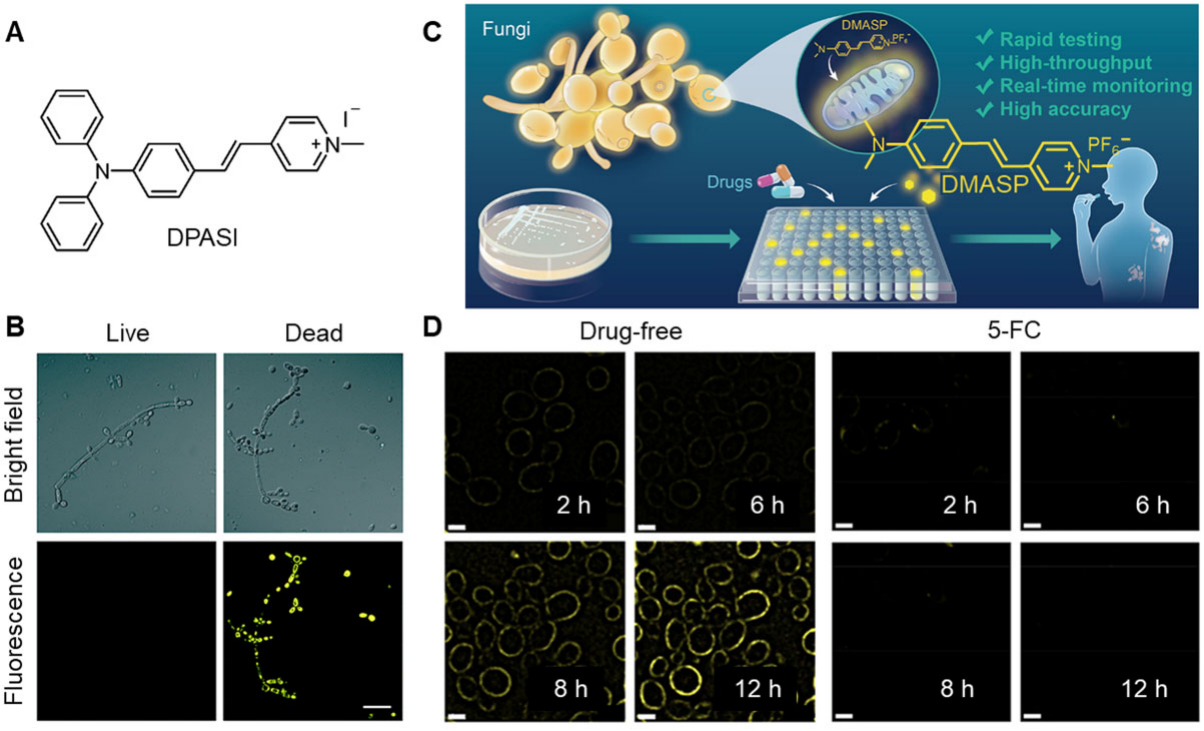
AIE materials for detection of fungal viability and susceptibility. (**A**) Molecular structure of DPASI. (**B**) Confocal laser scanning microscope (CLSM) images of C. albicans stained with DPASI. Reproduced from Ref. [67] with permission of the Royal Society of Chemistry and the Chinese Chemical Society, © 2020. (**C**) Schematic illustration of rapid and high-throughput testing of antifungal susceptibility using an AIEgen-based analytical system. (**D**) Structure illumination microscopy (SIM) fluorescence images of drug-free and 5-flucytosine (5-FC) treated C. albicans suspensions versus time. Scale bar = 6 μm. Reproduced from Ref. [68] with permission of Elsevier, © 2022.

Fungi, a type of eukaryotes, are pathogenic to humans [57]. It is reported that fungi-related infectious diseases have been emerging as an important public health problem [58]. Evaluation of fungal viability and antifungal susceptibility was considered as key factors for the inspection and treatment of fungi-related infection in clinic [59, 60]. Previous methods widely used for fungal viability test are time-consuming and labor-intensive, involving counting the CFUs after incubation, and using methylene blue to stain and count dead cells [61–65]. In comparison, fluorescence imaging offers a more rapid and accurate method for fungi detection with the privileges of easier operation and higher sensitivity [66]. In recent years, AIEgens that could stain fungi have been widely developed [67, 68]. An AIE-active probe DPASI has been designed by Zheng’s group for sensing fungal viability (Fig. 5A). This molecule is water-dispersible and could selectively stain dead *Candida albicans* in a wash-free manner within 5 min. With the help of DPASI, live and dead *C. albicans* could be easily differentiated in fluorescence channel (Fig. 5B). The fast imaging of dead *C. albicans* cells with impaired cell wall could be ascribed to the efficient binding of DPASI with mitochondria. Therefore, this probe provides a facile and promising platform not only for the rapid detection of fungal viability but also for the accurate screening of new antifungal drugs, which would greatly contribute to the fungal research.

Another AIE probe DMASP was developed for anti-fungal susceptibility testing (AFST), which has been verified to be the most valuable way to determine the *in vitro* effectiveness of antifungal agents in clinic (Fig. 5C). Mitochondria-specific DMASP can monitor the change of mitochondrial membrane potential (MMP) caused by non-specific cation influx of the hyperpolarized membrane. In the AFST of azoles (e.g. fluconazole and itraconazole), elevated MMP can be reflected by the enhancement of fluorescence intensity, indicating the increased accumulation of positive-charged DMASP into mitochondria. As a contrast, antifungal drugs 5-flucytosine (5-FC) displayed no fluorescence in DMASP-based AFST because their rapid and efficient killing effect to fungi weakened the binding of DMASP to fungi (Fig. 5D). What’s more, DMASP-based AFST system could differentiate fungal resistance among clinical fungal isolates via evaluating the time resolved fluorescence intensity. Compared with the time-consuming clinical standard AFSTs, this method benefits the advantages of easy operation, real-time monitoring and quantitative analysis, displaying great contribution to the detection of possible drug-resistant fungal strains and the precise use of antimicrobials against fungal diseases.

### AIE biomaterials for viruses detection

**Figure 6.**
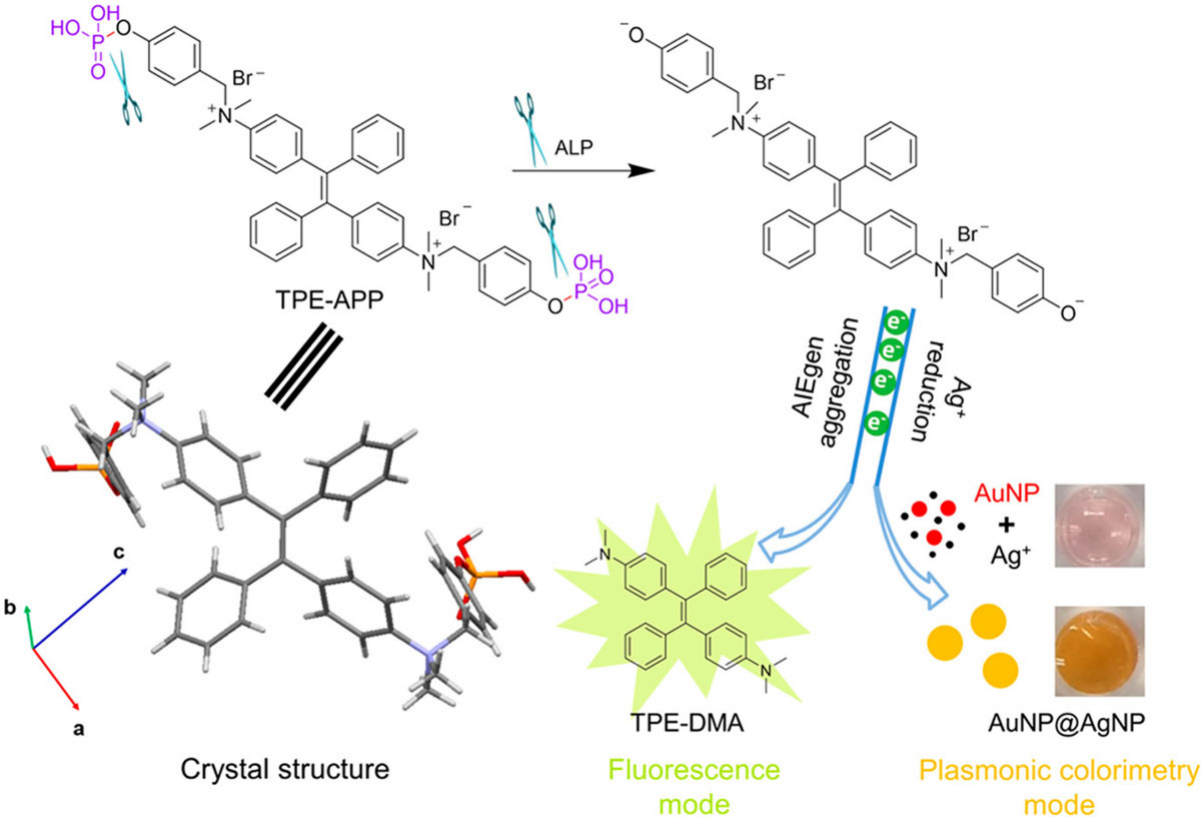
AIE Materials for virus detection. Schematic illustration of fluorescent and plasmonic colorimetric dual-modality for virus detection based on a multifunctional AIEgen TPE-APP. Reproduced from Ref. [74] with permission of American Chemical Society, © 2018.

Rapid, sensitive and accurate detection of highly contagious viruses (e.g. SARS-CoV-2) is urgently demanded for the prevention and control of virus pandemic [69, 70]. The advantages of AIEgen, i.e. luminosity, photobleaching resistance and biocompatibility promote their applications in ultrasensitive detection of viruses [71–73]. Xiong *et al*. [74] demonstrated a dual-modality readout immunoassay platform based on AIEgen for detecting of viruses. The mechanism of dual-mode detection of virus is shown in Fig. 6. A water-soluble multifunctional AIEgen (TPE-APP) with an enzymatic cleavage group was designed. The probe can be hydrolyzed by alkaline phosphatase to form water-insoluble TPE-DMA aggregate and a highly redox species. On one hand, the TPE-DMA aggregate is intensively fluorescent. One the other hand, the redox species could reduce silver ion to generate a silver nanoshell on the surface of AuNP (AuNP@AgNP) that leads to the blue shift of the localized surface plasmonic resonance peak of AuNPs, displaying a pronounced color change from red to yellow and further to brown, which can be differentiated by naked eyes. By further taking advantage of effective immunomagnetic enrichment, enterovirus 71 (EV71) virus, as an example, can be specifically detected with a limit of detection concentration down to 1.4 copies/μl under fluorescence modality and in a broad range from 1.3 × 10^3^–2.5 × 10^6^ copies/μl with naked eyes. Most importantly, EV71 virions in 24 real clinical samples are successfully diagnosed with 100% accuracy. Compared to the gold standard polymerase chain reaction assay, this immunoassay platform is not only much more convenient and cheaper, but also show excellent quantitatively capability, high sensitivity and strong anti-interference ability, paving great prospect for handy preliminary screening and high accuracy in clinical diagnosis of viruses.

**Figure 7.**
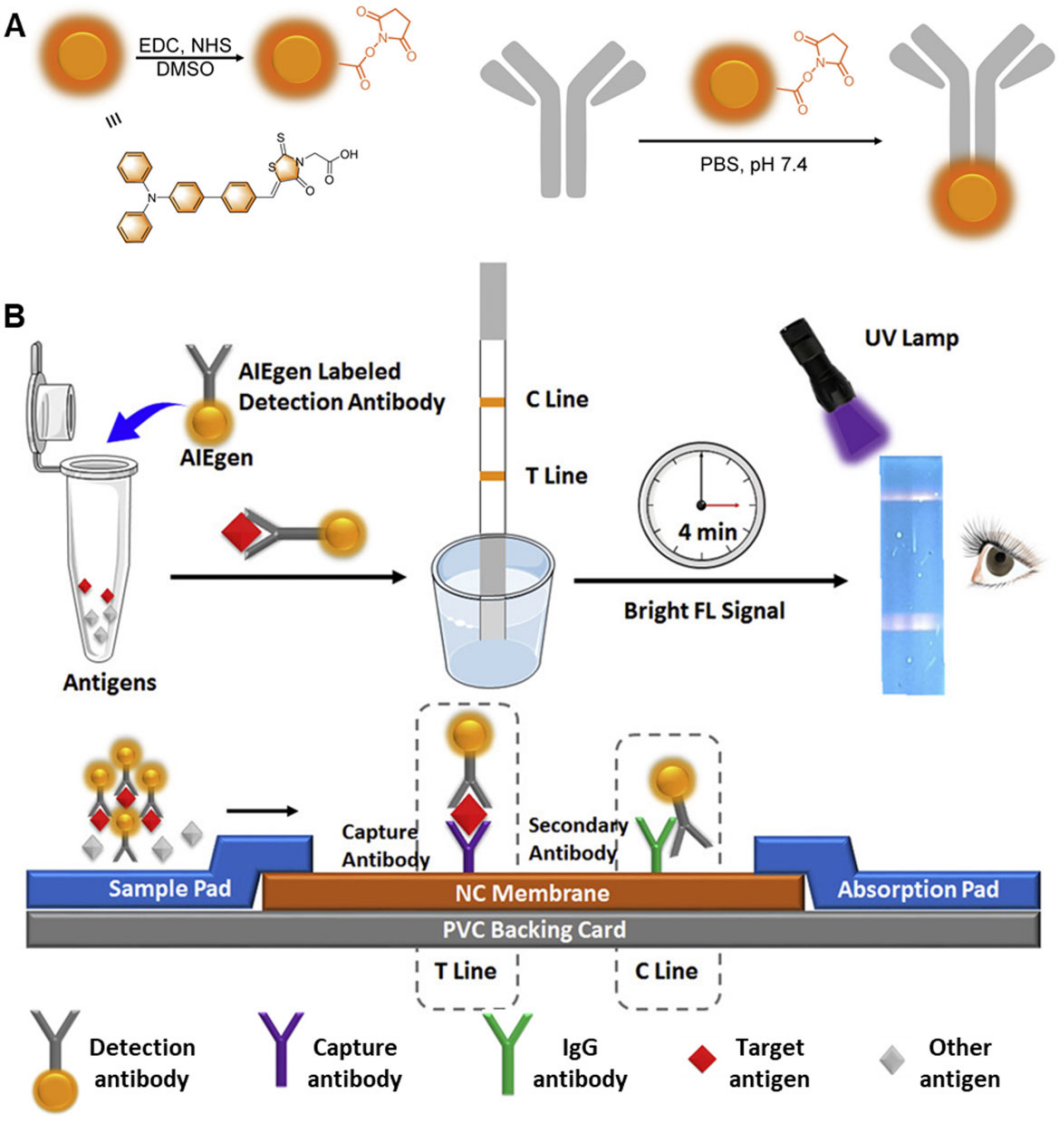
AIE Materials for detection of viral protein. (**A**) Schematic of the preparation of NHS modified AIEgen and the antibody labeling with AIEgen. (**B**) Schematic of the general test strip platform based on AIEgens as a reporter for SARS-CoV-2 antigen detection. Reproduced from Ref. [79] with permission of American Chemical Society, © 2018.

In addition, AIEgens have also been applied in viral proteins detection. Viral antigen-based detection could be developed as a complementary screening strategy for early diagnosis of SARS-CoV-2 infection. SARS-CoV-2 consists of four structural proteins, known as spike (S), envelope (E), membrane (M) and nucleocapsid (N) proteins [75]. Among them, N protein has been identified as one of the best early diagnostic targets and could be detected before the appearance of antibody in serum [76, 77]. Furthermore, the receptor-binding domain (RBD) of S protein could be directly tested without virus lysis, which could act as a suitable diagnostic epitope [78]. Recently, Zhang *et al*. [79] developed a wearable lateral flow test strip based on AIEgens for rapid detection of SARS-CoV-2 protein (Fig. 7). AIEgens-labeled antibody is prepared by reaction between N-hydroxysuccinimide (NHS) activated AIEgen and antibody (Fig. 7A). The structure and the working mechanism of the strip were shown in Fig. 7B. In general, for the detection antibody without binding reaction with the target antigen, it passed through the T line and bound to the C line. After 4 min, bright fluorescent signal could be easily observed under 365 nm irradiation. Due to the high brightness and anti-bleaching ability, the detection limit can be as low as 6.9 ng/ml for RBD protein and 7.2 ng/ml for N protein, respectively. Compared with the antigen test based on colloidal gold or fluorescein isothiocyanate, this testing platform provides higher sensitivity, better specificity and stronger anti-inference ability. Therefore, this AIEgen-based strip presented excellent testing performance and thus could be built as a promising platform for rapid and accurate virus detection during the pandemic.

## AIE biomaterials for pathogens inactivation

### AIE biomaterials for bacteria inactivation

Upon the detection of pathogenic bacteria, it is especially important to kill the pathogenic bacteria efficiently. In this section, different methods based on AIE biomaterials for bacterial inactivation will be presented, including AIE antibiotics, PDT and PTT [80–83].

#### Antibiotics derivatives with AIE characteristics

Since penicillin was discovered in 1928, antibiotics have been widely used all over the world [8, 84]. Due to antibiotic abuse, multidrug-resistant bacteria can evade antibiotics and thus have become a great threat to human health. Hence, production of new antibiotics is highly desirable [85, 86]. Pharmacologists usually focus on the structure–activity relationships of antibiotics to enhance their activity, with their optical properties ignored. In fact, investigating their photophysical properties is of great significance to understand their working mechanism [87]. It is reported that fluorescent antibiotic and its derivatives have great potential to become a new research tool to fight antibiotic resistance.

**Figure 8.**
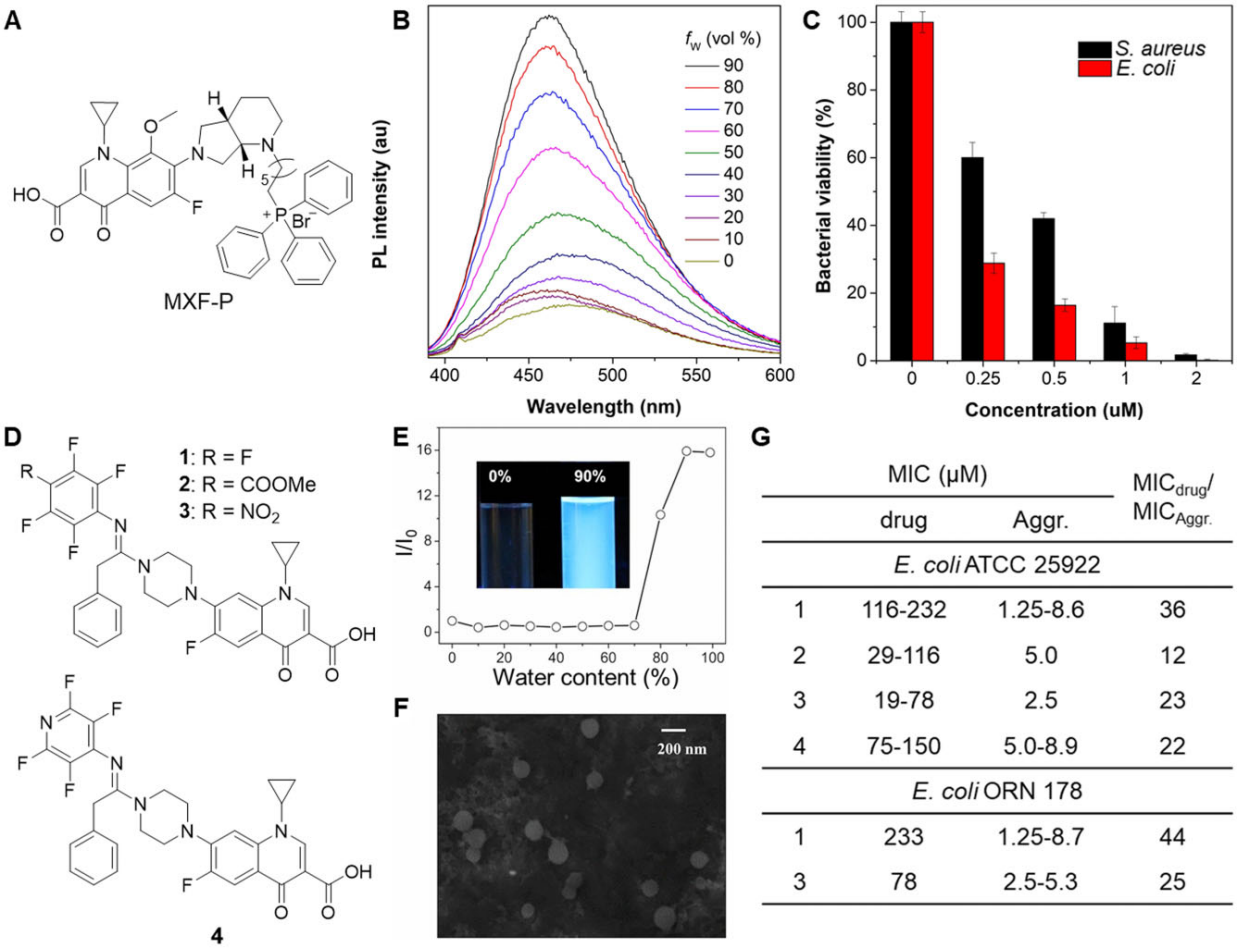
Antibiotics derivatives with AIE characteristics. (**A**) Chemical structure of moxifloxacin derivative MXF-P. (**B**) PL spectra of MXF-P in water/DMSO mixtures with different water fraction. (**C**) Antibacterial activity against bacteria of MXF-P at different concentrations. Reproduced from Ref. [88] with permission of Elsevier, © 2022. (**D**) Chemical structures of ciprofloxacin derivatives with AIE property. (**E**) Plot of relative emission intensity at 457 nm for 1 against water content in water/MeCN mixtures. *I*_0_: emission intensity at 0 vol% water. (**F**) SEM image of 1 prepared from 90 vol% water/MeCN. (**G**) MIC of the ciprofloxacin derivatives against *E. coli*. Reproduced from Ref. [90] with permission of National Academy of Sciences, © 2017.

Recently, Wang *et al*. [88, 89] studied the AIE properties of several FDA-approved fluoroquinolone antibiotics (norfloxacin (NOR), levofloxacin (LEV), and moxifloxacin hydrochloride (MXF-HCl)). After a systematical structure–activity relationship study, MXF modified with a triphenylphosphonium (MXF-P) was successfully prepared with AIE feature and comparable antibacterial activity to MXF (Fig. 8A and B), which could be applied for rapid imaging of bacteria. Furthermore, MXF-P exhibited admirable antibacterial activity *in vitro* and *in vivo* by inhibition of bacterial division and destruction of the envelope structure (Fig. 8C). Similarly, Xie *et al*. [90] developed a series of AIE active ciprofloxacin derivatives for bacterial theranostics based on pure nanodrugs strategy (Fig. 8D–F). The nanoaggregates of ciprofloxacin derivatives showed excellent fluorescent properties with up to 11% quantum yield. Then, the antibacterial activities of the nanoaggregates were compared with molecular drugs. As shown in Fig. 8G, the nanoaggregate generally showed much better antibacterial activity than the soluble form with much lower minimal inhibit concentrations (MICs), demonstrating that nanodrug could enhance antibacterial activity of the molecular drug. These works provide an innovative strategy for new multifunctional drug discovery based on new use of old drug principle.

#### Natural compounds with AIE property and photodynamic activity

**Figure 9.**
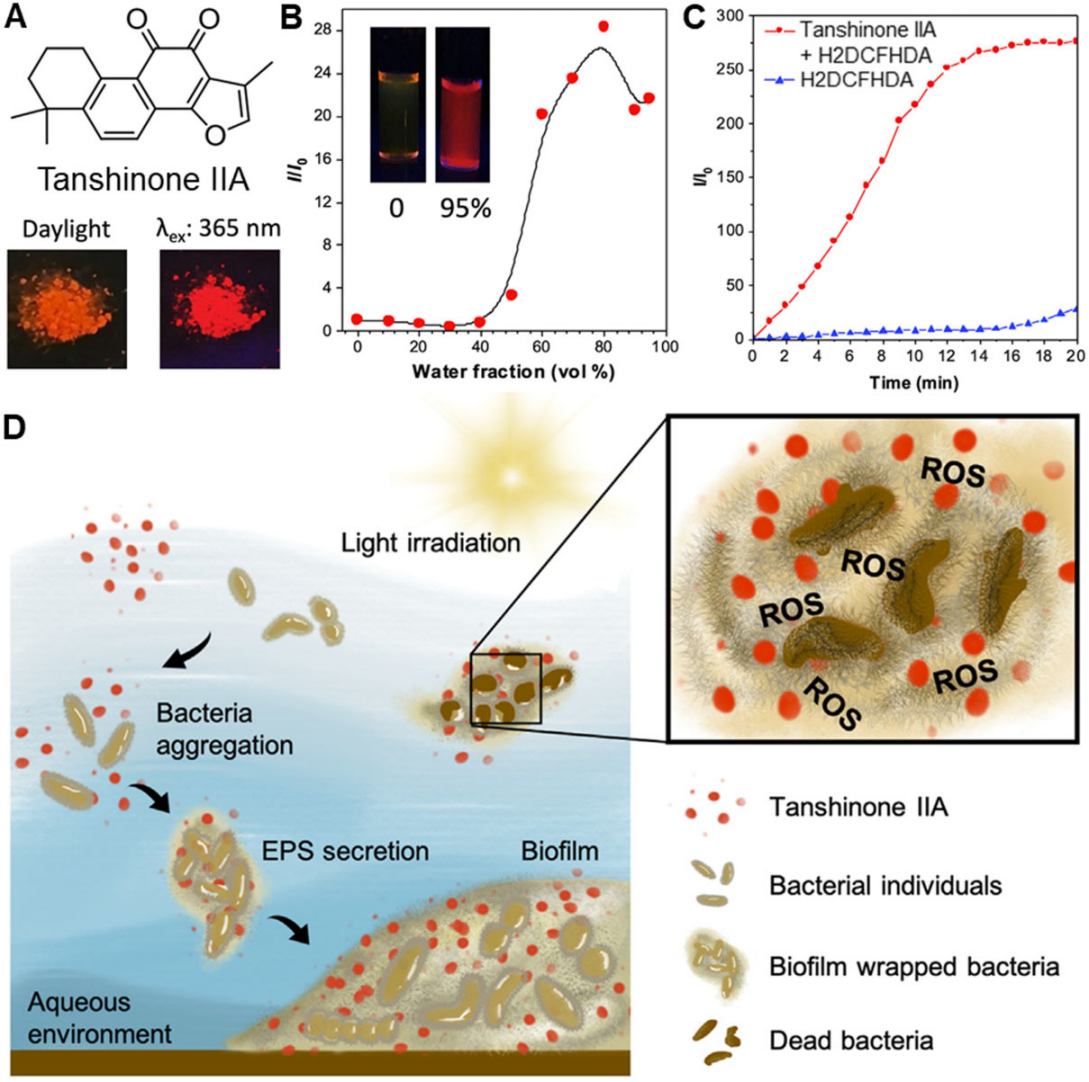
Natural product with AIE property and photodynamic activity. (**A**) Chemical structure of tanshinone IIa and its photographs under daylight and 365 nm irradiation. (**B**) The plot of the emission maximum and the relative emission intensity (*I*/*I*_0_) versus the composition of the DMSO/water mixtures of tanshinone IIA. Inset: fluorescence photographs of tanshinone IIa in DMSO and in DMSO/water mixtures with 95% water fraction taken under 365 nm UV irradiation. (**C**) ROS generation of tanshinone IIa upon white-light irradiation. (**D**) Schematic illustration of tanshinone IIa for simultaneous targeting and photodynamic eradication of bacterial biofilms. Reproduced from Ref. [92] with permission of Elsevier, © 2022.

In addition to modification of antibiotics, some multifunctional natural products with AIE properties have been applied for bacteria inactivation [91]. Lately, Michelle *et al*. [92] report an AIE active natural compound Tanshinone IIA and investigated its application in photodynamic eradication of biofilm (Fig. 9). Tanshinone IIA is a traditional Chinese medicine and its chemical structure is shown in Fig. 9A. The photophysical properties were well characterized (Fig. 9B). Tanshinone IIA could efficiently generate ROS under white light irradiation (Fig. 9C). Furthermore, it was found that Tanshinone IIA could specifically stain bacterial biofilms, remarkably promote bacterial aggregation and photodynamic eradication of biofilm (Fig. 9D).

#### AIE biomaterials for photodynamic inactivation of bacteria

**Figure 10.**
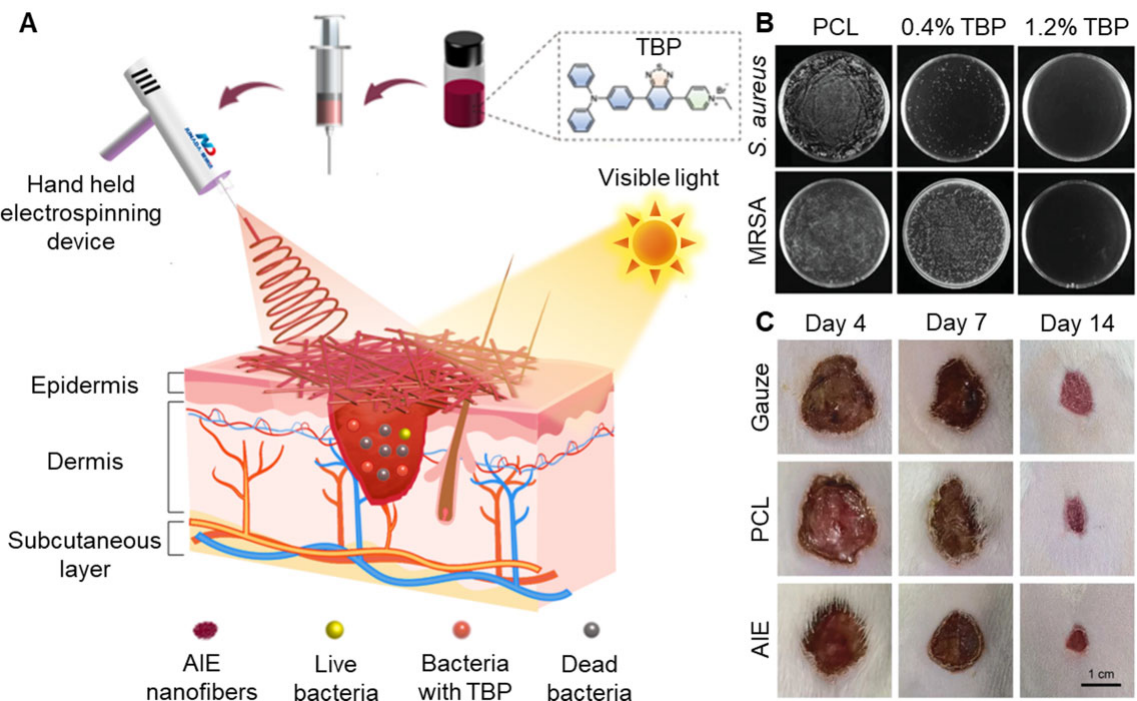
AIE materials for bacterial PDT. (**A**) Schematic illustration of electrospinning AIEgen TBP-incorporated antibacterial dressing. (**B**) Photos of *S. aureus* and MRSA treated with different TBP contents of TBP/PCL nanofibers upon white light irradiation (16 mW/cm^2^) for 20 min.(**C**) photographs of the appearance of the wounds after different treatments. Reproduced from Ref. [95] with permission of Wiley-VCH, © 2022.

Rapid wound dressing and effective antibacterial therapy are of extreme importance for skin wounds treatment in emergency [93, 94]. By combing the advantages of *in situ* electrospinning and AIE PSs, Zhao *et al*. [95] developed a feasible and rapid strategy for wound infection treatment of surgery or accident injuries by *in situ* depositing AIEgen-based nanofibrous dressing (Fig. 10A). In their approach, AIEgens (TBP)-incorporated poly(ε-caprolactone) (PCL) solution could be readily electrospun by a handheld electrospinning device to form the nanofibrous dressing, which was closely and well adhesive to the wound surface. *In vitro* (Fig. 10B) and *in vivo* (Fig. 10C) experiments demonstrated that the AIE nanofibrous dressing exhibited excellent antibacterial activities and greatly promoted the wound healing process. Thus, the strategy of *in situ* deposition of AIE nanofibrous dressing via handheld electrospinning device can provide personalized therapies for emergency wounds with the advantages of convenience, versatility and universality.

In addition to explore the applications of AIE PS in different scenarios, researchers also devote a lot to elucidate the relationship of structure and function relationship of antibacterial agents to achieve accurate and efficient antibiosis [96]. Kang *et al*. [97] designed a family of positively charged PSs with AIE feature. By adjusting the Clog*P* value (3–5), these positively charged AIEgens could selectively stain G+ bacteria without any extra targeting groups. Benefiting from the excellent specificity to G+ bacteria, effective ROS generation ability, and bright NIR emission, these AIEgens were successfully utilized for the selective photodynamic theranostics of *S. aureus*. This successful example on structure–activity relationship study could provide guidance for the further exploration of novel antibacterial theranostic agents.

#### AIE biomaterials for photothermal inactivation of bacteria

**Figure 11.**
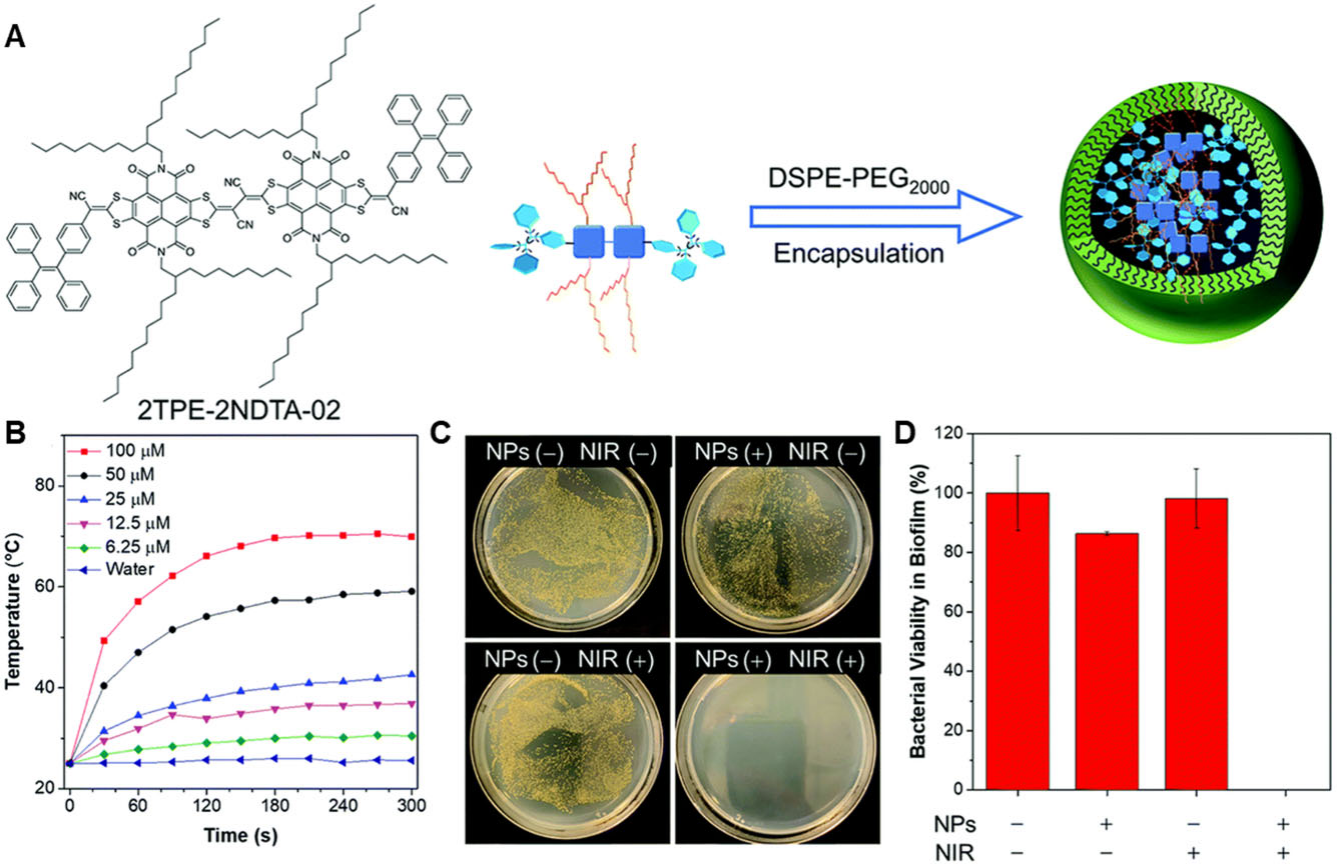
AIE materials for bacterial PTT. (**A**) Schematic illustrations of fabrication of TN NPs by encapsulating DSPE-PEG_2000_ with 2TPE-2NDTA-02. (**B**) Photothermal conversion behavior of TN NPs in aqueous solution at different concentrations (0–100 μM) under 808 nm laser irradiation. (**C**) Photographs of bacteria colonies grown on NB agar. (**D**) Relative bacterial viability in the treated biofilm of each group. Reproduced from Ref. [102] with permission of the Royal Society of Chemistry, © 2021.

Biofilm-related infections, such as dental plaque and bone implant, have brought great suffering to patients and heavy financial burden to society [98, 99]. Bacterial biofilm is a unified community where bacteria are encapsulated by the matrix, leading to the resistance to antibiotics [100]. PTT has been widely studied as one of the most attractive strategies for combating bacteria with its advantages on light-controllability and hardly no drug resistance [101]. Recently, He *et al*. [102] report a highly efficient photothermal nanoparticle (TN NP) to eradicate biofilms (Fig. 11). TN NPs were prepared by encapsulation of NIR-absorbing photothermal AIEgens (2TPE-2NDTA-02) with DSPE-PEG_2000_ (Fig. 11A). By taking advantage of active intramolecular motions in the aggregate state and enhanced molar absorptivity, TN NPs showed high ability in photothermal conversion and excellent photobleaching resistance. Under 808 nm NIR laser irradiation, the temperatures of the aqueous solutions elevated with time and saturated within 3 min (Fig. 11B). The prepared NPs can effectively eliminate mature *S. aureus* biofilms upon NIR laser by destruction of the adjacent bacteria and deactivation of the adhesive components (Fig. 11C−D). Therefore, PTT is one of the most promising potential candidates for the clinical treatment of biofilm in the future.

#### AIE materials for bacterial photodynamic/photothermal synergistic therapy

**Figure 12.**
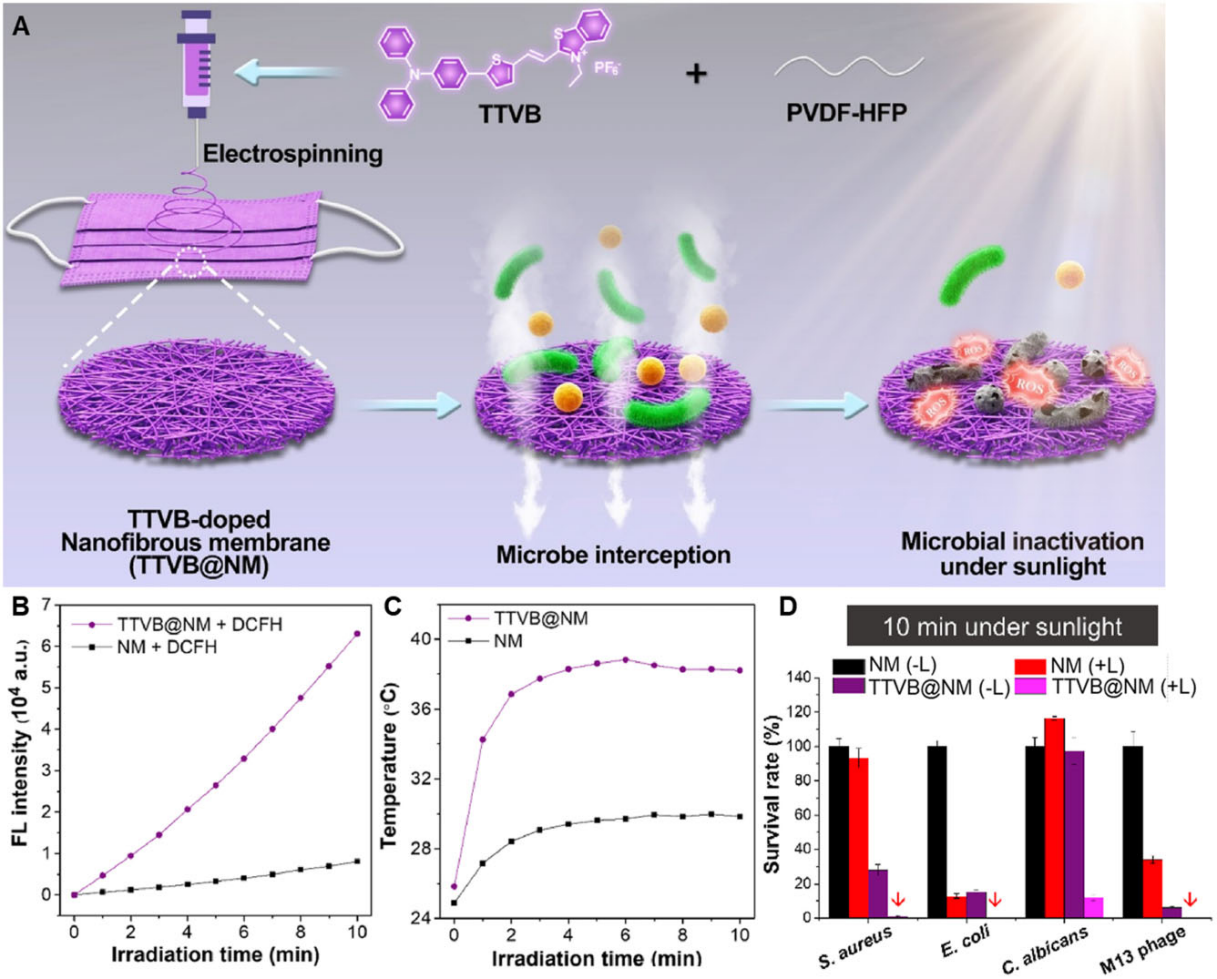
AIE Materials for bacterial photodynamic/photothermal synergistic therapy. (**A**) Schematic illustration of the preparation of TTVB-loaded nanofibrous membrane (TTVB@NM) through electrospinning for microbial inactivation under sunlight. (**B**) ROS generation of TTVB@NM under the irradiation of simulated sunlight. (**C**) The photothermal conversion performance of NM and TTVB@NM under the irradiation of simulated sunlight. (**D**) Survival rates of different microbes after sunbathing (red arrows: antimicrobial efficacy is around 100%). Reproduced from Ref. [105] with permission of Elsevier, © 2021.

Some AIEgens have been developed with synergistic PDT and PTT effect [103, 104]. For instance, Li *et al*. [105] developed an AIEgens-loaded mask with sunlight-triggered photodynamic/photothermal anti-bacterial functions by utilizing AIEgen based nanofibrous membrane (TTVB@NM) (Fig. 12A). Under simulated sunlight irradiation, TTVB@NM exhibited efficient ROS generation and moderate photothermal conversion performance (Fig. 12B and C). Furthermore, TTVB@NM was covered on a medical mask and displayed efficient antimicrobial activity against pathogenic aerosols (Fig. 12D). It is notable that the filtration efficiency and air permeability of the TTVB@NM coated mask showed comparable even better performance than that of other reported antimicrobial wearable materials.

### AIE biomaterials for fungi inactivation

Fungus is a common microorganism widely found in nature, which can lead to various severe infections, like fungal keratitis, fungal dermatosis etc. [106, 107]. The treatment of fungi-related infectious diseases is still challenging because current antifungal drugs suffer drug resistance and side effects. Recently, some AIEgens with PDT activity are reported as promising candidates for the treatment of fungal infections with the advantages of negligible drug resistance, high spatiotemporal accuracy, and low side effects [108].

**Figure 13.**
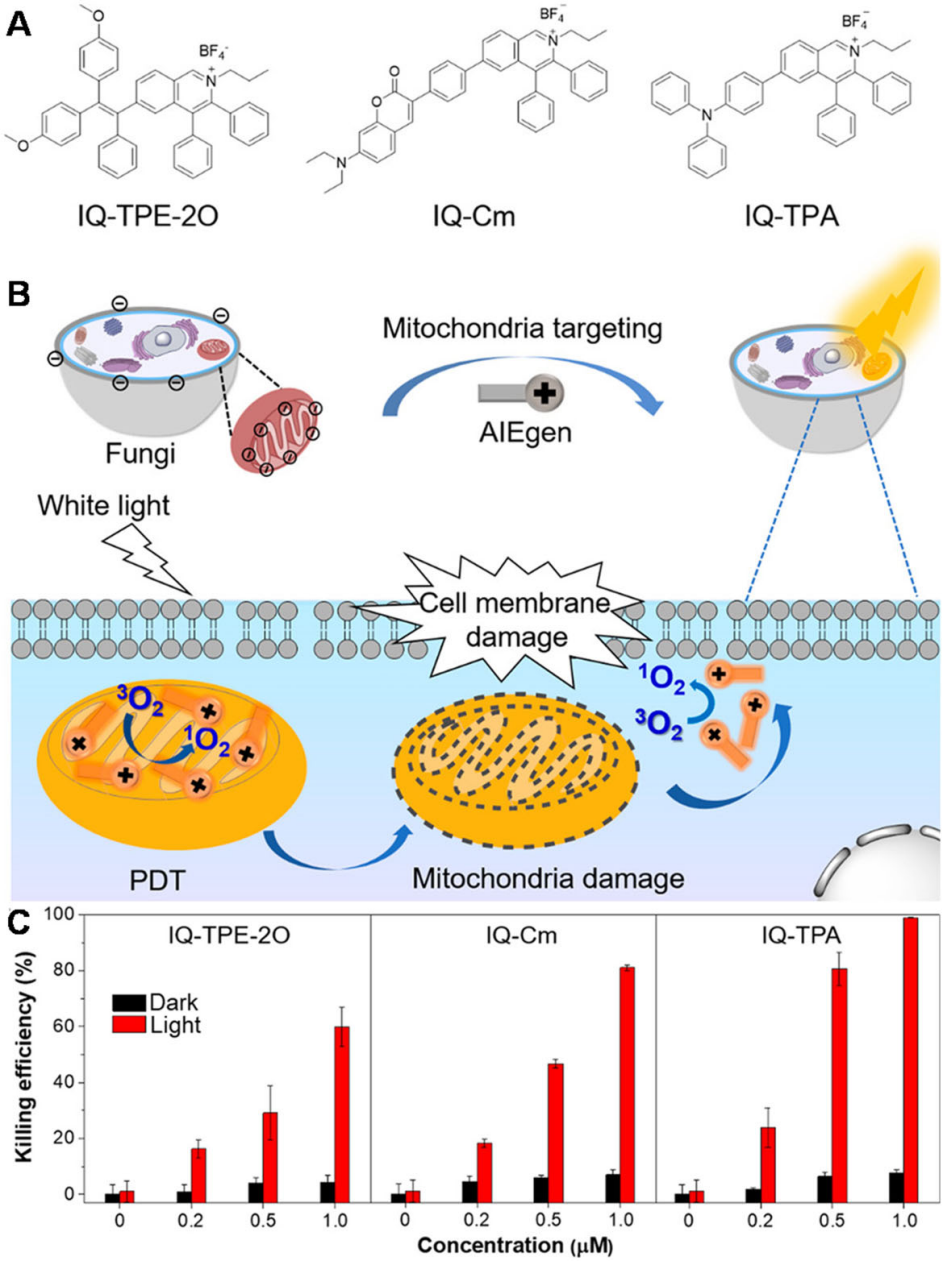
AIE Materials for selective photodynamic killing of fungi. (**A**) Molecular structure and (**B**) schematic illustration of AIEgens for selective mitochondria-specific PDT of fungi. (**C**) Antifungal activity of AIEgens toward *C. albicans* at different concentrations in the dark and under white light irradiation. Reproduced from Ref. [111] with permission of American Chemical Society, © 2021.

Fungal keratitis is a widespread disease which would cause corneal tissue injury and sight-threatening complication [106]. Mitochondria is considered as an ideal target for fungi inactivation due to its important role in fungi morphogenesis virulence and drug resistance [109]. As such, selectively targeting fungal mitochondria is a valuable approach for fungi treatment with low side effects [110]. Zhou *et al*. [111] developed a series of mitochondria-specific isoquinolinium (IQ)-based AIEgens (IQ-TPE-2O, IQ-Cm and IQ-TPA) for selectively photodynamic killing of fungi and effective treatment of keratitis (Fig. 13A). Driven by the intrinsic discrepancy in surface membrane potential between fungi and mammalian cells and negative mitochondrial MMP, these cationic AIEgens designed with proper hydrophobicity were expected to preferentially accumulate in the mitochondria of fungi over mammalian cells and cause mitochondrial disruption by PDT effect (Fig. 13B). The results indicate that all three AIEgens, especially IQ-TPA, exhibit excellent antifungal activity for *in vivo* treatment, which is much better than the clinically used PSs rose bengal (Fig. 13C).

### AIE biomaterials for viruses inactivation

Besides bacterium and fungus, virus is another great threat to public health. There are numerous barriers in the prevention and control of virus-related pandemics due to the lack of specific drugs. Personal protective equipment (PPE), like masks and protective suits, is a key factor to inhibit the spread of virus, preventing healthy people especially frontline healthcare doctors and nurses from virus infection [112]. Due to poor self-virucidal capabilities of traditional PPEs, viruses accumulate on the fibers of PPEs over long-term usage, leading to wearers’ infection. In addition, conventional PPEs are usually disposable with limited service life [113]. Further, the improper disposal of PPEs is likely to cause cross-contamination [114]. Therefore, there is an emergency to develop self-antiviral PPEs with long-term usable and reusable characteristics.

**Figure 14.**
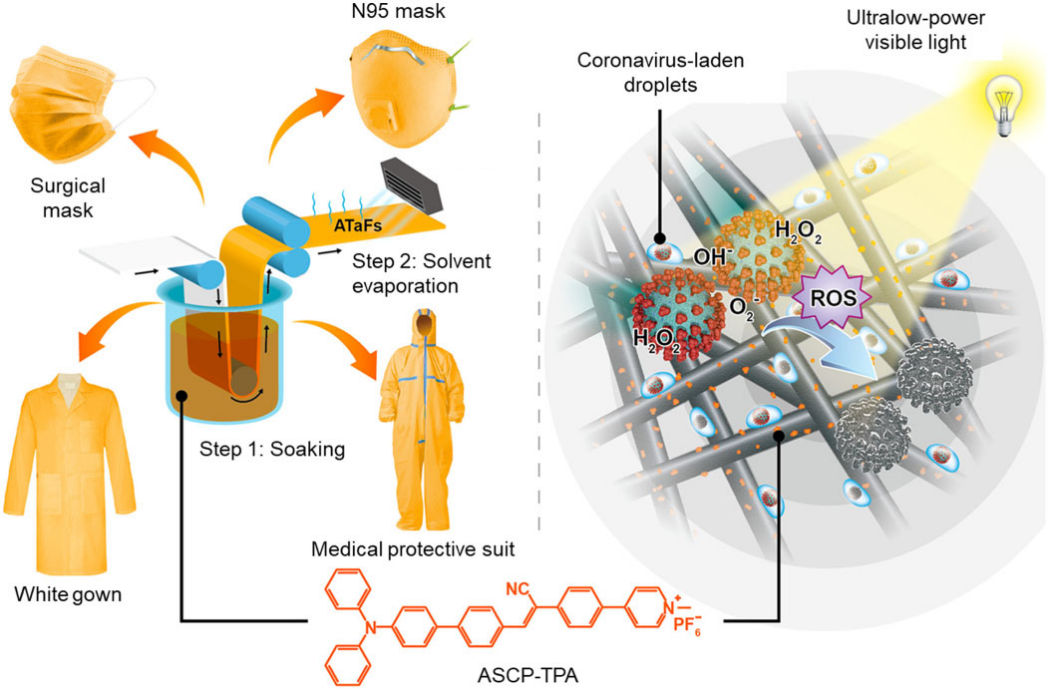
AIE Materials for self-antiviral PPEs. schematic diagram of the preparation of ASCP-TPA-attached fabrics (ATaFs) and various ATaF-based PPEs (left) and the photodynamic inactivation principle of ATaFs against coronavirus upon ultralow-power light irradiation (3.0 mW/cm^2^) (right). reproduced from Ref. [115] with permission of American Chemical Society, © 2021.

A series of AIE PS based PPEs with real-time self-antiviral capabilities has been successfully reported recently (Fig. 14) [115]. For instance, the AIEgen ASCP-TPA was synthesized with the advantages of facile synthesis, excellent biocompatibility and superior ROS generation ability. ASCP-TPA-attached fabrics could efficiently inactivate SARS-CoV-2 within 10 min under ultralow-power visible light irradiation (3.0 mW/cm^2^). Moreover, the modified fabrics not only showed low toxicity to normal cell but also displayed highly durable anti-virus properties despite of 100 washings or exposure to office light for 2 weeks. This strategy shows a great potential to fight against SARS-CoV-2 or other airborne pathogens and to improve global PPE supply shortages.

**Figure 15.**
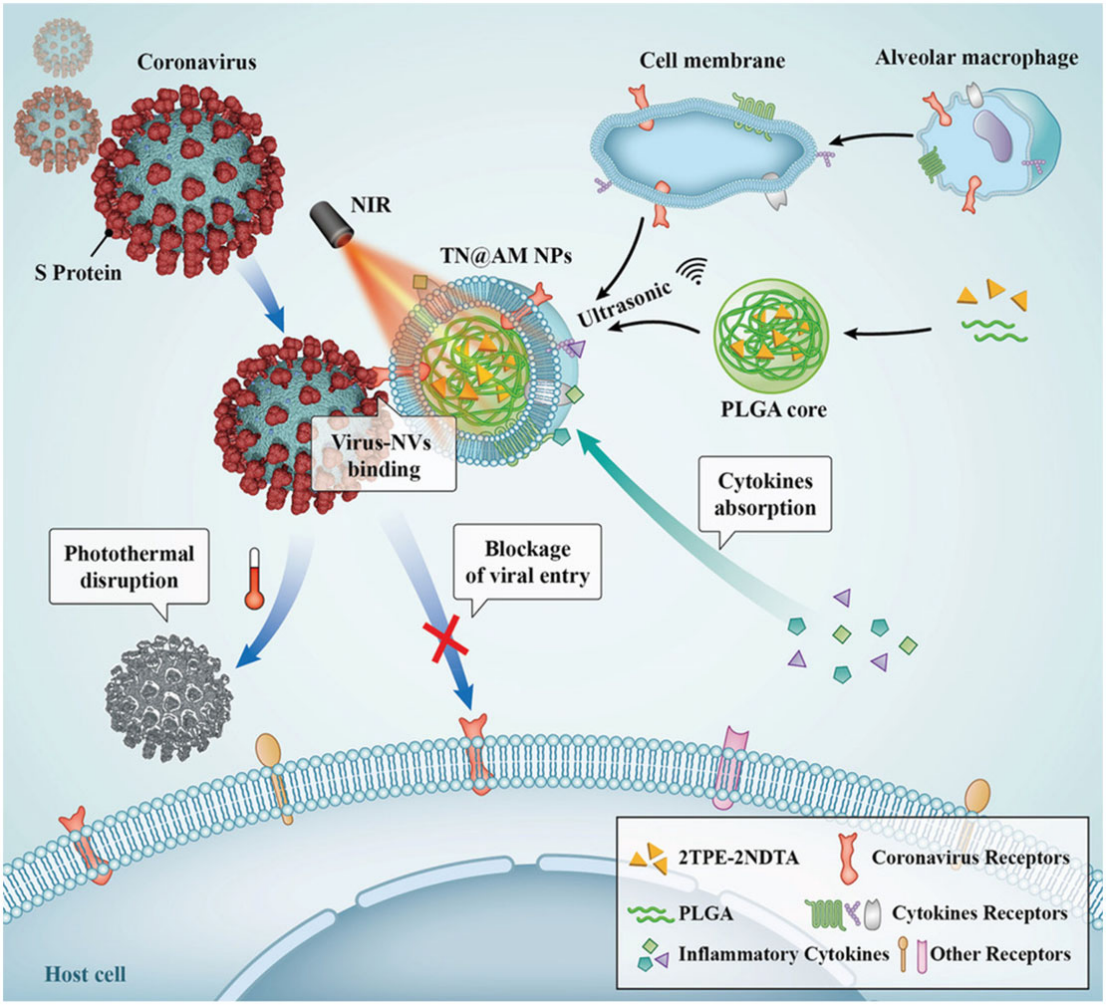
AIE Materials for photothermal disruption of virus. Schematic illustration of multifunctional AM-like NPs for coronavirus cellular entry blockage, virus photothermal disruption, and inflammatory cytokines absorption. Reproduced from Ref. [117] with permission of Wiley-VCH, © 2021.

Multifunctional AIE biomaterials with antiviral and anti-inflammatory abilities is highly desirable. To date, cell membrane-coated nanoparticles (NPs) have emerged as a biomimetic nanomedicine platform for treatment of different diseases [116]. Based on this bioinspired strategy, Li *et al*. [117] developed a type of multifunctional photothermal NPs coated by alveolar macrophage (AM) membrane to kill coronavirus. The AM membranes endow NPs with function as the coronavirus receptor and multiple cytokine receptors for coronavirus cellular entry and various proinflammatory cytokine binding, respectively, to keep the virus away from their host targets. After treatment of a surrogate mouse model of COVID-19 caused by murine hepatitis virus A-59 (MHV-A529) with these multifunctional AM-bioinspired NPs under NIR irradiation, not only the virus burden and cytokine levels in lungs decreased but also tissue damage and inflammation were relieved and improved. Moreover, the viral transmission and infection progress can be restricted after PTT. This work provides an ideal strategy to fabricate novel multifunctional bioinspired NPs with cell membrane-coating method for the effective treatment of COVID-19 (Fig. 15).

In addition to photothermal reagents, AIEgens are also utilized for viral photodynamic inactivation. Recently, Wu *et al*. [118] reported a new AIEgen (DTTPB) for highly efficient photodynamic inactivation of human coronavirus. DTTPB with a hydrophilic head and two hydrophobic tails can selectively bind the envelope of human coronavirus. Under low power white light irradiation (9 mW/cm^2^), DTTPB showed excellent antiviral effect on human coronavirus. These AIEgens could target the viral phospholipid bilayers or protein covering viral capsid via the lipophilic cations of IQ, thus destroy the viral structure under white light irradiation.

## Summary and outlook

In the past 20 years, AIEgens-based applications have been extensively developed, especially in biomedical field, including biosensing, bioimaging, cancer theranostics, microbe inactivation etc. This review aims to highlight the latest advances of AIEgens-based biomaterials for theranostics of bacteria, fungi and viruses. Based on the nature of these three pathogens, AIEgens are well modified and thus endowed with the capability of pathogen differentiation and selective imaging. Integrated with chemotherapeutic activity or phototherapeutic activity, multifunctional AIE biomaterials are developed for efficient pathogen inactivation.

Although AIEgens-based antimicrobial theranostics have made a remarkable progress, there are still much room for improvement. (i) Visualization of interactions of bacteria–bacteria, microbe–bacteria plays an important role in understanding the corresponding physiological processes. To date, several NIR dyes have been developed for covalent binding with bacteria based on metabolic labeling strategy [119]. As a result, the endocytosis of bacteria, the interaction of gut microbiota and other dynamic processes could be real-time monitored. (ii) Artificial intelligent (AI) assisted pathogen differentiation can significantly improve the accuracy, rapidity and sensitivity, which is highly suitable for high-throughput assay. (iii) The back-to-basics principle of new uses of old drugs has emerged as a fruitful basis for the discovery of new AIE drugs [120]. Thus, some AIE-active natural compounds with advantages of biocompatibility, biodegradability and intrinsic pharmacological activity, have been developed for various applications. (iv) Biosafety is a frequently discussed topic, which is an essential factor to advance the clinical application. To date, the excretion of AIE NPs in hepatobiliary and gastrointestinal pathway of non-human primates and rodents has been investigated [121]. Also, the distribution of AIE NPs in different tissue was studied, revealing that AIE NPs have low passive permeability to tumor regions [122]. More studies on AIE biomaterials, such as bio-distribution and pharmacokinetics, need to be conducted in future.
